# Activation of transposable elements and genetic instability during long-term culture of the human fungal pathogen *Candida albicans*

**DOI:** 10.1007/s10522-019-09809-2

**Published:** 2019-04-15

**Authors:** Leszek Potocki, Ewelina Kuna, Kamila Filip, Beata Kasprzyk, Anna Lewinska, Maciej Wnuk

**Affiliations:** 1 Department of Genetics, Faculty of Biotechnology, University of Rzeszow, Pigonia 1, 35-310 Rzeszow, Poland; 2 Department of Cell Biochemistry, Faculty of Biotechnology, University of Rzeszow, Pigonia 1, 35-310 Rzeszow, Poland

**Keywords:** *Candida albicans*, Long-term culture, Transposon activity, Karyotype profiling, DNA breaks, Stress inductors

## Abstract

It has been repeatedly reported that transposable elements (TE) become active and/or mobile in the genomes of replicatively and stress-induced senescent mammalian cells. However, the biological role of senescence-associated transposon activation and its occurrence and relevance in other eukaryotic cells remain to be elucidated. In the present study, *Candida albicans*, a prevalent opportunistic fungal pathogen in humans, was used to analyze changes in gene copy number of selected TE, namely Cirt2, Moa and Cmut1 during long-term culture (up to 90 days). The effects of stress stimuli (fluconazole, hydrogen peroxide, hypochlorite) and ploidy state (haploid, diploid, tetraploid cells) were also considered. An increase in copy number of Cirt2 and Moa was the most accented in tetraploid cells after 90 days of culture that was accompanied by changes in karyotype patterns and slightly more limited growth rate compared to haploid and diploid cells. Stress stimuli did not potentiate TE activity. Elevation in chromosomal DNA breaks was also observed during long-term culture of cells of different ploidy, however this was not correlated with increased TE activity. Our results suggest that increased TE activity may promote genomic diversity and plasticity, and cellular heterogeneity during long-term culture of *C. albicans* cells.

## Introduction

The budding yeast *Saccharomyces cerevisiae* has become the most popular unicellular and genetically tractable aging model system, playing a key role in the discovery of Sir2, TOR, RAS, adenylate cyclase, PKA and S6 kinase as conserved modulators of longevity in eukaryotes (Kaeberlein 2010; Longo et al. 2012). In general, there are two assays for yeast aging, namely the evaluation of replicative lifespan (RLS) and chronological lifespan (CLS) (Kaeberlein 2010; Longo et al. 2012). RLS measures the replicative potential of the cell, i.e. the number of daughter cells produced by a mother cell before senescence (Mortimer and Johnston 1959), whereas CLS measures the survival of a non-dividing population in a stationary culture (Fabrizio and Longo 2003). More recently, a new distinctive microbial model for cellular aging has been established, namely *Candida albicans*, a polymorphic fungus and a prevalent opportunistic fungal pathogen in humans (Fu et al. 2008). Both RLS as well as CLS assays have been adapted to *C. albicans* aging studies (Fu et al. 2008; Chen et al. 2012; Lin and Austriaco 2014). *C. albicans* can switch between two distinct morphological states, namely a yeast-like form (blastospore) and a filamentous form (hyphae) that can be modulated by nutrient composition in a culture medium, pH or temperature. Smaller replicatively young yeast form (daughters) and replicatively old hyphae (mothers) can be separated by centrifugation on a sucrose gradient that, in contrast to *S. cerevisiae* RLS, allows for more efficient large-scale isolation of old cells and may facilitate biochemical characterization and genomics/proteomics studies of cellular aging (Fu et al. 2008). Similarly to *S. cerevisiae*, replicatively old *C. albicans* cells have been shown to accumulate glycogen and oxidatively damaged proteins (Fu et al. 2008). Moreover, deletion of the *SIR2* gene resulted in decreased RLS, while insertion of an extra copy of *SIR2* extended RLS that indicate that Sir2 is also a regulator of cellular aging in *C. albicans* (Fu et al. 2008). It has been reported that CLS of *C. albicans* could be also extended by reducing the concentration of glucose from 2% to 0.5% in synthetic complete (SC) medium (calorie restriction conditions) that has been previously observed during chronological aging in *S. cerevisiae* (Chen et al. 2012). Moreover, *C albicans* as a Crabtree negative fungus that prefers respiration to fermentation even in the presence of glucose may be considered as a good model for providing complimentary comparisons to aging and calorie restriction studies in a Crabtree positive *S. cerevisiae* (Lin and Austriaco 2014).

In general, two classes of transposable elements (TEs) can be distinguished, namely class I elements (copy-and-paste retrotransposons) that utilize reverse transcribed RNA intermediates to produce copies of themselves and class II elements (cut-and-paste DNA transposons) that excise from a donor site to reintegrate elsewhere in the genome (Wicker et al. 2007; Burns 2017). It has been suggested that more than half of human DNA is comprised of interspersed repeats resulting from replicative copy and paste events of retrotransposons (Burns and Boeke 2012). Altered expression of transposable elements can drive mutations in tumorigenesis and can be considered as a hallmark of cancer (Burns 2017). More recently, activation of transposable elements has been also documented in replicatively and stress-induced senescent human cells as well as during normal aging in mammalian somatic tissues (De Cecco et al. 2013a, b; Colombo et al. 2018; De Cecco et al. 2019). However, little is known about the biological function(s) of age-associated increase in TE activity and related mechanisms. Age-mediated changes in the mobilomes of lower eukaryotes and non-mammalian systems, especially in well-established model organisms, and their consequences also have been poorly addressed.

The aim of the present study was to investigate the changes in the copy number of selected TEs (Cirt2, Moa and Cmut1) during long-term culture of *C. albicans* cells of different ploidy (haploid, diploid and tetraploid cells) in control conditions as well as after treatment with stress stimuli (fluconazole, hydrogen peroxide, hypochlorite), and their effects on growth rate, cell viability, karyotype patterns and genetic instability. We have developed an experimental protocol for a long-term culture of *C. albicans* cells at a high density in a rich and fresh YPD medium that mimicked the survival of a non-dividing population in a stationary culture in synthetic complete (SC) medium (Fabrizio and Longo 2003). However, as a spent medium has been replaced by a fresh one every 2 days of 90 days of culture, the impact of acidification of the culture medium, the phenomenon of accumulation of acetic acid in SC spent medium during chronological aging in yeast (Burtner et al. 2009), on *C. albicans* cell viability was minimized as well as starvation and related nutritional stress responses were limited. We have shown for the first time that TE activity is elevated during long-term culture of *C. albicans* cells that is accompanied by changes in karyotype profiles and may in turn promote genomic diversity and cellular heterogeneity as an adaptive response.

## Materials and methods

### Strains and culture conditions

The following *Candida albicans* strains were used: 302 (haploid), SC5314 (diploid) and T15 (FH6, tetraploid but trisomic for chromosomes 2/3 with multiple copies of chromosome 5L) (Selmecki et al. 2008). The strains were a generous gift from Prof. Judith Berman (Department of Molecular Microbiology and Biotechnology, Tel Aviv University, Israel).

*C. albicans* cells from one single colony were routinely cultured on liquid yeast extract peptone dextrose (YPD) medium (1% w/v Difco Yeast Extract, 2% w/v Difco Yeast Bacto-Peptone, 2% w/v dextrose) (BD Biosciences, Sparks, MD, USA) with shaking at 28 °C. For long-term cultures during stress conditions, several stress stimuli were considered, namely fluconazole (100 ng/ml, Sigma-Aldrich, Poznan, Poland), hydrogen peroxide (2 mM, Sigma-Aldrich, Poznan, Poland) and sodium hypochlorite (5 mM, Sigma-Aldrich, Poznan, Poland). The concentrations of stress inductors were selected on the basis of IC_50_ values. For long-term cultures, cells at the logarithmic phase of growth were suspended at 1 × 10^8^ cells/ml in YPD medium with or without stress stimuli and cultured with shaking at 28 °C. During the first 4 days of long-term culture, fresh solutions of tested agents were added every 12 h and then stress stimuli were removed and culture was continued for up to 90 days with medium change every 48 h. After 14, 28 and 90 days of culture, samples were taken for the evaluation of prolonged effects of stress stimuli after their removal from the medium.

### Growth rate and cell viability

After 14, 28 and 90 days of culture, growth rate was analyzed as previously described (Lewinska et al. 2011). Briefly, cells were removed, washed, suspended in YPD medium (a total volume of 150 μl with working concentration of 5 × 10^6^ cells/ml per well was considered) and cultured in a 96-well format incubator with shaking at 28 °C for 8 h. Cell growth was monitored turbidimetrically at 600 nm in a Tecan microplate reader every 1 h during a 8 h period.

After 14, 28 and 90 days of culture, cell viability was analyzed as previously described (Lewinska et al. 2014a). Briefly, LIVE/DEAD^®^ Yeast Viability Kit (Thermo Fisher Scientific, Warsaw, Poland) was used according to the manufacturer’s instructions. Cells were washed and stained with a mixture of FUN^®^ 1 and Calcofluor^®^ White M2R and inspected under an Olympus BX61 fluorescence microscope equipped with a DP72 CCD camera and Olympus CellF software. A total of 200 cells were used for the analysis.

### Cell morphology analysis using imaging flow cytometry

*Candida* cell morphology (shape) was investigated using Amnis^®^ FlowSight^®^ imaging flow cytometer and IDEAS software version 6.2.187.0 (Merck Millipore, Warsaw, Poland). Two subpopulations of cells were considered, namely spherical cells (circularity) and non-spherical cells (non-circularity). Representative histograms and cell images are presented. The parameter circularity object was analyzed and normalized frequency was plotted against circularity object. 5000 cells per sample triplicate were analyzed. The percentage of cells of non-spherical cell subpopulation is presented.

### Cell size analysis

Digital images of *Candida* cells were captured using an Olympus BX61 fluorescence microscope equipped with a DP72 CCD camera and Olympus CellF software (Olympus,Warsaw, Poland). ImageJ software (http://rsbweb.nih.gov/ij/) was used to analyze the cell size. Cell size was expressed as arbitrary units [a.u.].

### DNA content analysis

*Candida* cells were fixed and stained as comprehensively described elsewhwere (Potocki et al. 2019). Some minor modifications were provided, namely fixed cells were treated with 5 mg/ml proteinase K for better staining performance. Briefly, for DNA visualization, cells were counterstained with a drop of mounting medium containing 4′,6′-diamino-2-phenylindole (DAPI) (Cambio, Cambridge, UK) and then analyzed using an Olympus BX61 fluorescence microscope equipped with a DP72 CCD camera and Olympus CellF software (Olympus,Warsaw, Poland). The CCD capture conditions were as the following: exposure time 150 ms, 100x oil immersion objective. DAPI fluorescent signals were collected using DAPI filters (λ_ex_ = 345 nm, λ_em_ = 455). Fluorescence microscopy was adapted for DNA content analysis (Potocki et al. 2019). ImageJ software (http://rsbweb.nih.gov/ij/) was used to analyze the nuclear DNA content. DNA content was expressed as arbitrary units [a.u.].

### Karyotype profiling

DNA plugs were obtained using BIORAD CHEF Yeast Genomic DNA Plug Kit using a standard protocol (Selmecki et al. 2010) according to the manufacturer’s instructions. Contour clamped homogeneous electric field (CHEF)—pulsed-field gel electrophoresis (PFGE) separation of *C. albicans* chromosomes was performed on a 1% agarose gel in 0.8× TBE according to the manufacturer’s instructions using CHEF-DR^®^III Pulsed Field Electrophoresis System (Biorad, Warsaw, Poland) and the following conditions: 60 to 120 s switch, 6 V × cm^−1^, 120° angle for 36 h, followed by 120 to 300 s switch, 4.5 V × cm^−1^, 120° angle for 12 h. After CHEF-PFGE separation, *C. albicans* chromosomes were stained using ethidium bromide staining.

### Detection of transposable elements (TE): Southern blot analysis

First, several TE probes were created. The following oligonucleotide primer pairs for TE sequences, namely Cmut1, Cirt2 and Moa were designed using NCBI database:

- Cmut1: CAATAGCCACGACTTGCAGA, TCAGATAAATTGCTCGCATGA;
- Cirt2: TGGTCGATATGAAGTGGCTATG, ATTGTTGACACCCCACGACT;
- Moa: GTCGTGGAGTACGACGTTATCA, GTCGTGTAGTAGCAACACTTCG.

Amplification was performed in 50 µl aliquots of a solution containing 5 µl of 10× PCR buffer with MgCl_2_, 1.75 µl of 10 mM dNTPs, 300 nM primers and 3.5 U of Expand Long Template Enzyme Mix using Expand Long Template PCR System (Sigma-Aldrich, Poznan, Poland). The PCR reaction was performed in Mastercycler Eppendorf using the following conditions: initial denaturation at 94 °C for 2 min, followed by 10 cycles of denaturation at 94 °C for 10 s, annealing at 45 °C for 30 s, elongation at 68 °C for 2 min, and followed by 25 cycles of denaturation at 94 °C for 15 s, annealing at 52 °C for 30 s, elongation at 68 °C for 2 min and a final extension step at 68 °C for 7 min. Purified PCR products were labeled using the labeling kit DIG High Prime (Roche, Warsaw, Poland). Briefly, 1 μg DNA (a final volume of 16 μl) was denatured at 95 °C for 10 min and DNA was then labeled with 4 μl of digoxygenin-11-dUTP. Labeling reaction was performed overnight at 37 °C and then stopped by heating the samples at 65 °C for 10 min.

Second, transposable elements were detected using Southern blot analysis. After PFGE separation, *C. albicans* chromosomes were transferred onto a nylon membrane (Roche, Warsaw, Poland) by capillary transfer. Then, the membrane was hybridized to digoxigenin (DIG)-labeled TE-specific probes at 42 °C for 24 h and transposable elements (Cmut1, Cirt2, Moa) were detected using an alkaline phosphatase conjugated anti-DIG antibody. The chemiluminescence signal was detected using the substrate for alkaline phosphatase (DIG-High Prime DNA Labeling and Detection Starter kit II, Roche, Warsaw, Poland) and the G:BOX imaging system (Syngene, Cambridge, UK).

### qPCR-based analysis of TE copy number

The following primers were used:

- Cirt2: AATAATGGATGGGCATGGAA, CCAAGGTCCAAAGGCTGTAA;
- Cmut1: TAGCTCGGCCTCAACATTTCC, AGCGTTTTCTAGCACGAAAGC;
- Moa: GTCGTGGAGTACGACGTTATCA, GTCGTGTAGTAGCAACACTTCG.

qPCR reaction mixture contained: 2 µl of 300 ng DNA, 10 µl of Fast SYBR Green Master Mix (Roche, Warsaw, Poland), 1 µl of 20 µM each primer and ultrapure DNase/RNase-free water to a final volume of 20 μl. All qPCR reactions were performed in triplicate using Light Cycler^®^ 480 system (Roche, Warsaw, Poland). The following qPCR reaction conditions were: a single cycle at 95 °C for 10 min and 40 cycles of 15 s at 95 °C and 20 s at 62 °C. PCR products were then heated at 95 °C for 15 s, cooled at 60 °C for 1 min and heated at 95 °C for 15 s. The relative copy number of TE sequences was analyzed using the LightCycler^®^ 480 Software using the basic relative quantification according to beta-actin gene copy number.

### Analysis of chromosomal DNA breaks and replication intermediates (RIs)

For quantitative analysis of chromosomal DNA breaks and replication intermediates (RIs), single chromosome comet assay was used (Lewinska et al. 2014b). Preparation of agarose-embedded *C. albicans* DNA and PFGE separation of *C. albicans* DNA were performed as comprehensively described elsewhere (Lewinska et al. 2014b). After PFGE separation, *C. albicans* chromosomes were stained with ethidium bromide and bands were removed from the gel using a razor blade and single chromosome comet assay was conducted (Lewinska et al. 2014b). A total of 200 chromosomes per each sample strain triplicate were analyzed and the percentage of chromosomal DNA breaks and replication intermediates (RIs) were calculated as comprehensively described elsewhere (Adamczyk et al. 2016).

### Multivariate data analysis

All obtained data were subjected to multivariate analysis using the Hierarchical Cluster Analysis (HCA) using ClustVis, a web tool for visualizing clustering of multivariate data (BETA) (https://biit.cs.ut.ee/clustvis/) (Metsalu and Vilo 2015). The following options were considered: pre-processing option—no transformation, clustering distance for rows and columns—Pearson correlation, clustering methods for rows and columns—average, tree ordering for rows and columns—tightest cluster first. A heat map was generated on the basis of growth rate, TE copy number, karyotype patterns, chromosomal DNA breaks and replication intermediates.

### Statistical analysis

The mean values ± SD were calculated on the basis of at least three independent experiments. Box and whisker plots were also considered. Statistical significance was evaluated using GraphPad Prism 5 using one-way ANOVA and Dunnett’s test.

## Results

### Long-term culture-mediated changes in morphology, growth rate and cell viability

In the present study, we have used a model of long-term culture of *C. albicans* cells (up to 90 days) and started the culture at relatively high cell density (1 × 10^8^ cells/ml) to mimic stationary phase conditions during chronological aging (CA) in yeast that is measured by monitoring the survival of a non-dividing population (Longo et al. 2012; Hu et al. 2013). However, rich medium (YPD medium) was considered instead of a classical CA medium, namely synthetic complete (SC) medium and YPD medium was replaced by a fresh one every 48 h of culture to avoid starvation and related stress responses that are common during CA in yeast (Longo et al. 2012; Hu et al. 2013). Moreover, we decided to start analyzing the parameters of long-term culture experiment after 14 days of culture because culturing the yeast cells in rich YPD medium is characterized by extended postdiauxic phase (up to 1 week) during which cells continue to grow slowly (Werner-Washburne et al. 1996; Longo et al. 2012). *C. albicans* cells of different ploidy were used, namely haploid reference strain 302, diploid reference strain SC5314 and tetraploid reference strain T15 (Selmecki et al. 2008) and after 14, 28 and 90 days of culture, upon initial treatment with stress stimuli (fluconazole, hydrogen peroxide, hypochlorite) for 4 days, cells were taken for further analyses.

**Fig. 1:**
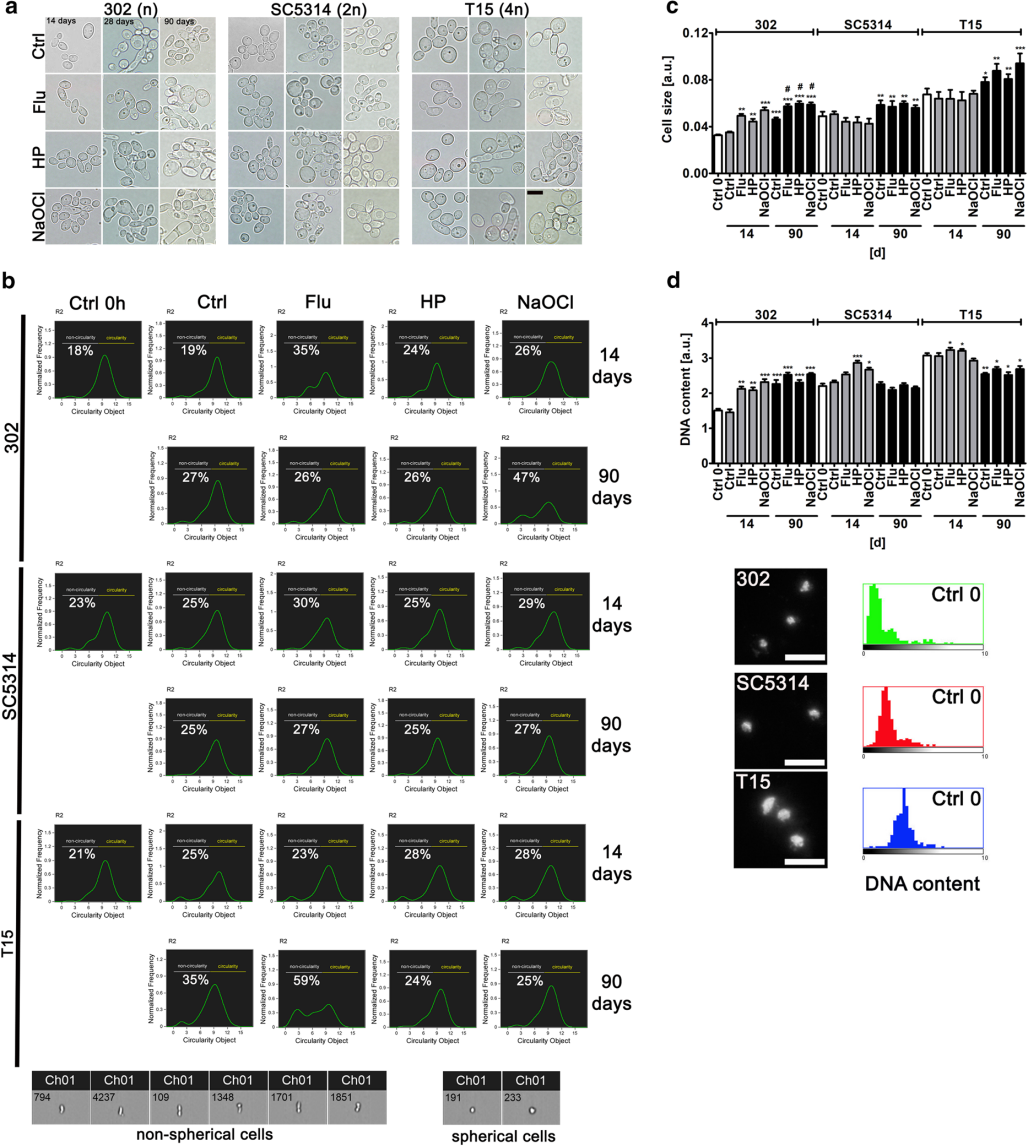
Long-term culture-mediated *Candida* cell morphology (**a**, **b**), cell size (**c**) and DNA content (**d**). **a** Cell morphology was analyzed using an Olympus BX61 fluorescence microscope equipped with a DP72 CCD camera and Olympus CellF software (Olympus). Representative microphotographs are shown. Scale bar 5 μm, objective 100×. **b** Cell morphology (here shape) was analyzed using Amnis^®^ FlowSight^®^ imaging flow cytometer and IDEAS software (Merck Millipore). Two subpopulations of cells were considered, namely spherical cells (circularity) and non-spherical cells (non-circularity). Representative histograms and cell images are presented. The parameter circularity object was analyzed and normalized frequency was plotted against circularity object. 5000 cells per sample triplicate were analyzed and the percentage of cells of non-spherical cell subpopulation is presented. **c** ImageJ software (http://rsbweb.nih.gov/ij/) was used to analyze the cell size. Cell size was expressed as arbitrary units [a.u.]. **d** Fluorescence microscopy was adapted for DNA content analysis. DNA was visualized using DAPI staining. Representative microphotographs and data distribution (histograms) are shown. Scale bars 10 μm, objective 100x. ImageJ software (http://rsbweb.nih.gov/ij/) was used to analyze the nuclear DNA content. DNA content was expressed as arbitrary units [a.u.]. Bars indicate SEM, n = 3. ***p < 0.001, **p < 0.01, *p < 0.05 compared to control conditions after 14 days of culture, ^#^*p* < 0.05 compared to control conditions after 90 days of culture (ANOVA and Dunnett’s a posteriori test). Ctrl, control conditions; Ctrl 0, control conditions at the logarithmic phase of growth (overnight culture); Flu, fluconazole; HP, hydrogen peroxide; NaOCl; sodium hypochlorite; 14, 14 days of culture; 28, 28 days of culture; 90, 90 days of culture; 302, haploid strain; SC5314, diploid strain; T15, tetraploid strain

In general, yeast cell morphology (budding form, single-celled blastospore) was observed when cells were cultured in YPD medium (Fig. 1a).

Filamentous (pseudohyphal and hyphal) forms were almost not noticed (Fig. 1a). Typical shapes (spherical to oval) and sizes (2–5 × 3–7 µm) of *C. albicans* cells were observed during long-term culture (Fig. 1a). However, after 90 days of culture, cells became more elongated as judged by morphology analysis using imaging flow cytometry (Fig. 1b). A small fraction of non-spherical cells occurred (Fig. 1b) that was especially accented in tetraploid cells (an increase of 10%) and in haploid cells (an increase of 8%) (Fig. 1b). After 14 days of culture in control conditions, cell morphology was not affected compared to cell morphology at logarithmic phase of growth (overnight culture, time 0) (Fig. 1b). The effects of stress stimuli on cell morphology were limited (Fig. 1b). Elevated fraction of non-spherical cell population was observed in hypochlorite-treated haploid cells and fluconazole-treated tetraploid cells after 90 days of culture, namely an increase of 21% and 36% was noticed, respectively (Fig. 1b). Moreover, ploidy-dependent cell size variation and DNA content analysis was considered (Fig. 1c, d). Of course, diploid and tetraploid cells were bigger than haploid cells after 14 days of culture in control conditions (Fig. 1c). After 90 days of culture, all three cell categories, namely haploid, diploid and tetraploid cells became bigger compared to 14 d culture in control conditions (Fig. 1c). Stress stimuli potentiated an increase in cell size of haploid cells both after 14 days as well as after 90 days of culture (Fig. 1c). As haploid strains of *C. albicans* are considered to be unstable and may autodiploidize (Hickman et al. 2013), we decided then to evaluate DNA content of haploid, diploid and tetraploid cells during long-term culture both in control conditions as well as upon stress agent stimulation (Fig. 1d). Of course, higher levels of DNA were observed in diploid and tetraploid cells compared to haploid cells after 14 days of culture (Fig. 1d). After 90 days of culture, DNA content of haploid cells was increased, whereas in tetraploid cells, DNA levels were decreased and DNA levels were not affected in diploid cells (Fig. 1d). Thus, it seems that DNA content of haploid, diploid and tetraploid cells is more or less comparable after 90 days of culture in control conditions. Perhaps, this may reflect a tendency of *Candida* cells to buffer their genome to be more or less diploid in term of DNA content (Hickman et al. 2013, 2015). This may be a form of adaptation during long-term culture. Moreover, all three stress stimuli potentiated an increase in DNA content of haploid cells after 14 day of culture compared to untreated control (Fig. 1d). There were no changes in cell size and DNA content of haploid, diploid and tetraploid cells between time categories of 0 and 14 days (Fig. 1c, d). One should remember that long-term culture-mediated cell size may not correlate with DNA content of yeast cells of different ploidy because during prolonged treatment and aging increased cell size may be associated with increased vacuole size.

**Fig. 2:**
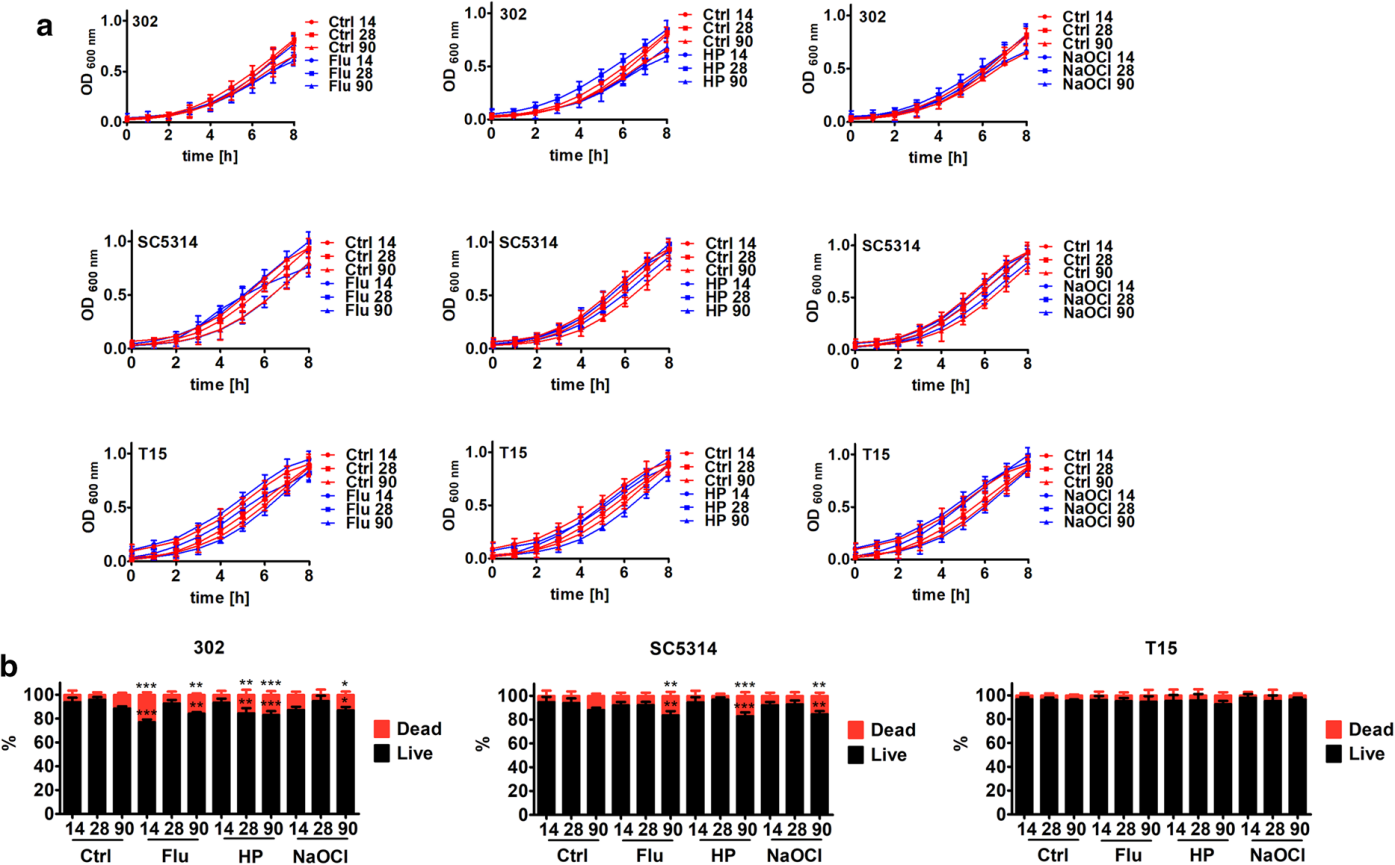
Long-term culture-mediated growth rate (**a**) and cell viability (**b**). **a** After 14, 28 and 90 days of culture, *C. albicans* growth was monitored turbidimetrically at 600 nm in a microplate reader every 2 h during a 8 h. **b** After 14, 28 and 90 days of culture, cell viability was estimated using a LIVE/DEAD^®^ Yeast Viability Kit using the standard protocol according to the manufacturer’s instructions. The percentage of live and dead cells is shown. Bars indicate SD, n = 200. ***p < 0.001, **p < 0.01, *p < 0.05 compared to control conditions after 14 days of culture (ANOVA and Dunnett’s a posteriori test). Ctrl, control conditions; Flu, fluconazole; HP, hydrogen peroxide; NaOCl; sodium hypochlorite; 14, 14 days of culture; 28, 28 days of culture; 90, 90 days of culture; 302, haploid strain; SC5314, diploid strain; T15, tetraploid strain

We have then addressed the question of whether growth rate and cell viability may be affected during long-term culture (Fig. 2).

There were no changes in growth rate of haploid 302 strain when grown up to 90 days both in control conditions and upon initial stimulation with stress agents for 4 days (Fig. 2a). In contrast, minor to moderate inhibitory effects were observed in diploid SC5314 and tetraploid T15 cells, respectively (Fig. 2a). Tetraploid cells were the most susceptible to long-term culture-mediated growth inhibition both in control conditions and upon treatment with stress agents (Fig. 2a). Cell viability of haploid, diploid and tetraploid strains was not affected during long-term culture in control conditions (Fig. 2b). Upon stimulation with stress agents, a slight increase in fraction of dead haploid and diploid cells was observed, especially after 90 days of culture (Fig. 2b). Similar effects were not noticed during long-term culture of tetraploid cells (Fig. 2b).

### Long-term culture-promoted activation of transposable elements and changes in karyotype patterns

**Fig. 3:**
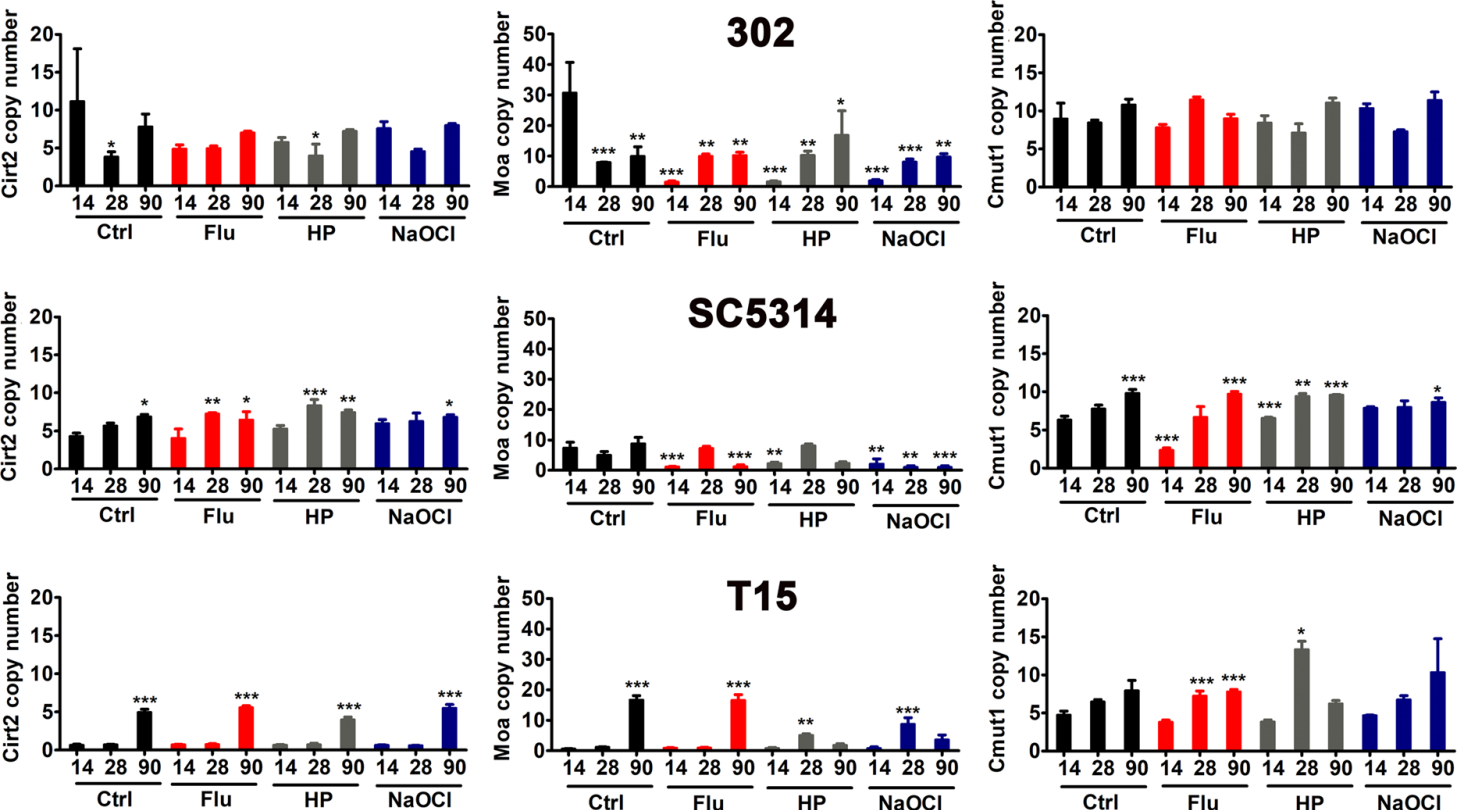
Long-term culture-mediated changes in the copy number of selected transposable elements, namely Cirt2, Moa and Cmut1. qPCR reactions were performed in triplicate using Light Cycler^®^ 480 system and relative copy number of TE sequences was analyzed using the LightCycler^®^ 480 Software using the basic relative quantification according to beta-actin gene copy number. Bars indicate SD, n = 3. ***p < 0.001, **p < 0.01, *p < 0.05 compared to control conditions after 14 days of culture (ANOVA and Dunnett’s a posteriori test). Ctrl, control conditions; Flu, fluconazole; HP, hydrogen peroxide; NaOCl; sodium hypochlorite; 14, 14 days of culture; 28, 28 days of culture; 90, 90 days of culture; 302, haploid strain; SC5314, diploid strain; T15, tetraploid strain

To study TE activity during long-term culture of *C. albicans* cells, we have selected three members from different TE groups, namely *C. albicans* transposon Cirt2 transposase (https://www.ncbi.nlm.nih.gov/nuccore/AF205929.1), *C. albicans* retrotransposon LTR Moa, LTR associated with the transposon Tca13 (https://www.ncbi.nlm.nih.gov/nuccore/AF180291.1) (Goodwin and Poulter 2000) and Cmut1 mutator-like TE (MULE) of DNA transposons (class II elements) with homology to chromosome R sequence (https://www.ncbi.nlm.nih.gov/nuccore/CP017630.1). For quantitative analysis of TE, copy number of Cirt2, Moa and Cmut1 was investigated using qPCR analysis (Fig. 3).

Increased copy number of Cirt2 and Moa was observed during long-term culture of tetraploid cells and to a lesser extent augmented copy number of Cirt2 and Cmut1 was noticed in diploid cells (Fig. 3). The effect of culture duration was much more accented than the effect of stress inductors (Fig. 3).

**Fig. 4:**
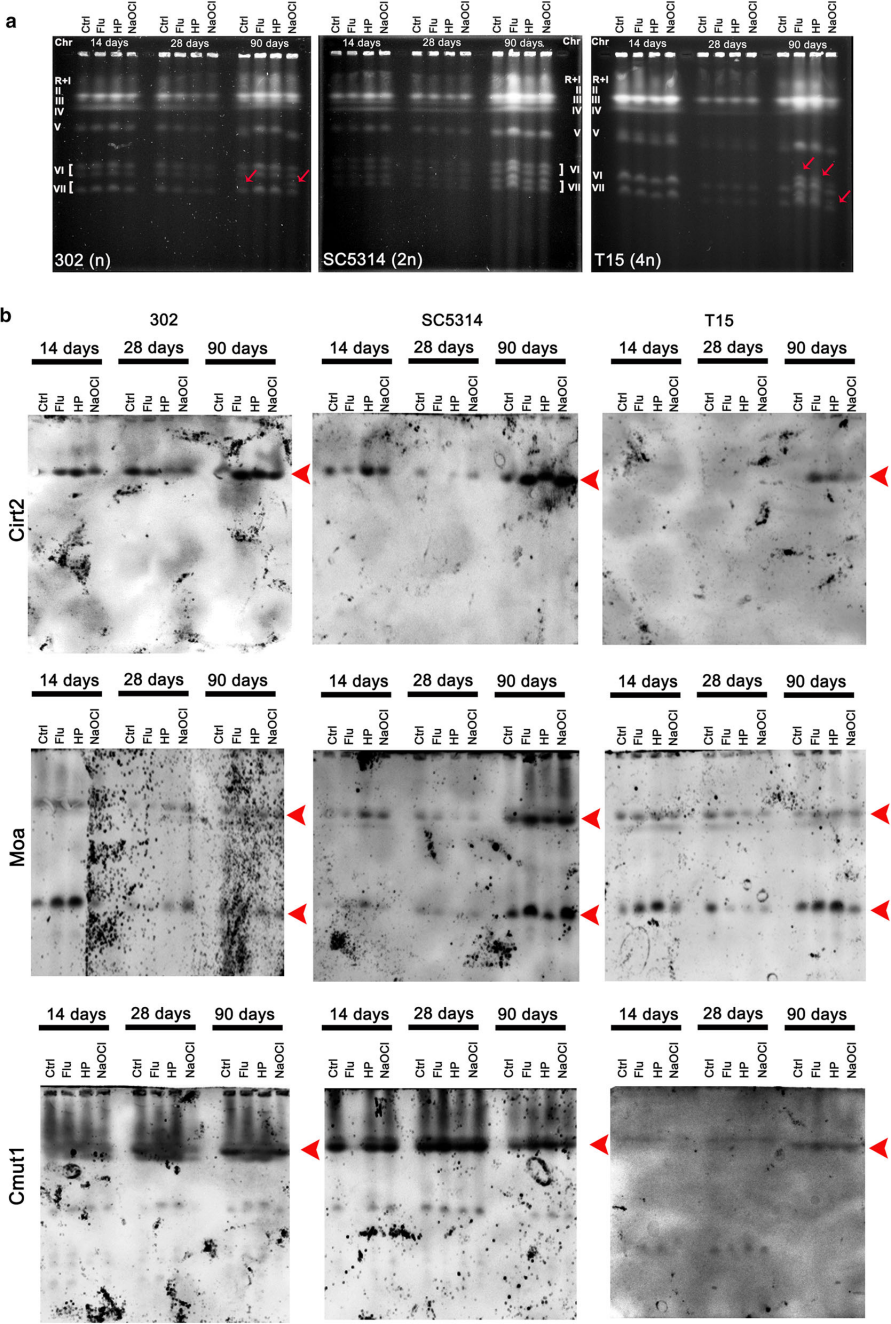
Long-term culture-mediated changes in karyotype patterns (**a**) and TE chromosomal localization (**b**). **a** Electrophoretic karyotyping (PFGE separation) of 302 haploid strain, SC5314 diploid strain and T15 tetraploid strain. Representative karyotype profiles are shown. Chromosomes were denoted as previously reported (Selmecki et al. 2005). Red arrows indicate additional chromosome bands that occurred after 90 days of culture. **b** Southern blot analysis of TE chromosomal localization. After PFGE separation, chromosomes were transferred onto a nylon membrane by capillary transfer, the membrane was hybridized to digoxigenin (DIG)-labeled TE-specific probes and transposable elements (Cmut1, Cirt2, Moa, red arrowheads) were detected using an alkaline phosphatase conjugated anti-DIG antibody. The chemiluminescence signal was detected using the substrate for alkaline phosphatase (Roche) and the G:BOX imaging system (Syngene). Ctrl, control conditions; Flu, fluconazole; HP, hydrogen peroxide; NaOCl; sodium hypochlorite; 14, 14 days of culture; 28, 28 days of culture; 90, 90 days of culture; 302, haploid strain; SC5314, diploid strain; T15, tetraploid strain

CHEF-PFGE separation was then considered for karyotype profiling during long-term culture (Fig. 4a).

According to PFGE results, we were able to distinguish from seven to eight separate chromosomes that is due to the fact that chromosome R and chromosome I may migrate together (Fig. 4a). Indeed, it is widely accepted that *C. albicans* cells have eight pairs of chromosomal homologs (Chibana et al. 2000), but one should also remember that *C. albicans* species are characterized by a high genomic diversity (Magee and Magee 1987; Chibana et al. 2000). There were no changes in karyotype patterns during long-term culture in control conditions and after treatment with stress stimuli in diploid cells (Fig. 4a). Thus, the genome of SC5314 cells at chromosome level may be considered stable and not prone to changes in chromosome number (Fig. 4a). In contrast, after 90 days of culture upon initial treatment with fluconazole, hydrogen peroxide and hypochlorite, additional chromosome bands were observed in tetraploid cells as well as to a lesser extent after 90 days of culture in control conditions and after treatment with hypochlorite in haploid cells (Fig. 4a, at chromosome VI and VII positions, red arrows). The most accented variability of karyotype profiles was correlated with the most pronounced increase in TE activity (here copy number) during long-term culture of tetraploid cells (Figs. 3, 4a). Thus, we decided then to analyze long-term culture mediated changes in chromosomal localization of TE (Fig. 4b). Cirt2 was detected on chromosome IV, Moa was detected on chromosomes I and VI and Cmut1 was detected on chromosome R (Fig. 4b, red arrowheads) that is in agreement with data provided at *Candida* genome database (www.candidagenome.org). However, no significant changes were found in their positions at chromosomes during long-term culture as judged by Southern blot analysis after CHEF-PFGE separation (Fig. 4b, red arrowheads). There were no correlations between the presence of additional bands (302 and T15 strains after 90 days of culture, Fig. 4a) and changes in TE chromosomal positions (Fig. 4b, red arrowheads). Southern blot analysis did not reveal increased levels of TE as already documented using qPCR analysis (Fig. 3). However, Southern blot is not as sensitive and reliable quantitative analysis of TE as qPCR. There are also some limitations concerning data normalization. Typically, an equal amount of cells is used for spheroplast preparation but in the next step, the quantity of agarose-embedded DNA is not assayed. Moreover, spheroplast preparation from older cells with thicker cell wall may be less efficient compared to younger cells. Thus, Southern blot analysis was used only for analyzing the changes in TE chromosomal localization (qualitative analysis).

### Long-term culture-induced chromosomal DNA breaks and replication intermediates

**Fig. 5:**
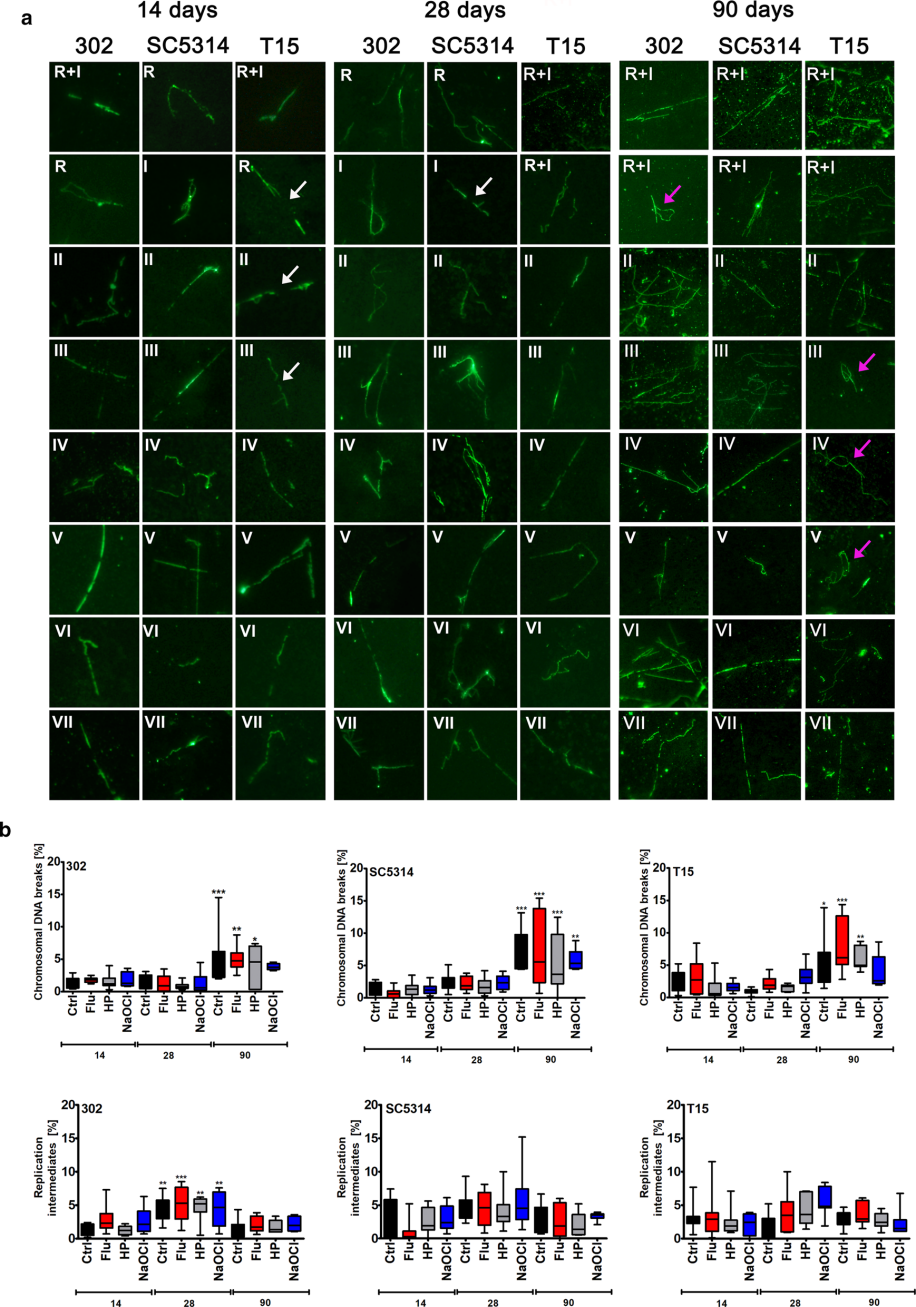
Long-term culture-induced chromosomal DNA breaks and replication intermediates. **a** After 14, 28 and 90 days of culture, chromosomal DNA breaks (white arrows) and replication intermediates (pink arrows) were revealed using single chromosome comet assay and an Olympus BX61 fluorescence microscope equipped with a DP72 CCD camera and Olympus CellF software. Briefly, *C. albicans* chromosomes were separated using PFGE and selected bands were cut using a razor blade. After electrophoresis, chromosomal DNA was stained with YOYO-1 stain solution (green). Representative microphotographs are shown. R+I; a joined fraction of chromosome R and chromosome I; II; chromosome II; III; chromosome III; IV; chromosome IV; V; chromosome V; VI; chromosome VI; VII; chromosome VII. **b** A total of 200 chromosomes per sample triplicate were analyzed and the percentages of chromosomal DNA breaks and replication intermediates were calculated. Box and whisker plots are shown, n = 3. ***p < 0.001, **p < 0.01, *p < 0.05 compared to control conditions after 14 days of culture (ANOVA and Dunnett’s a posteriori test). Ctrl, control conditions; Flu, fluconazole; HP, hydrogen peroxide; NaOCl; sodium hypochlorite; 14, 14 days of culture; 28, 28 days of culture; 90, 90 days of culture; 302, haploid strain; SC5314, diploid strain; T15, tetraploid strain

As increased TE activity may result in genetic instability, we have then considered single chromosome comet assay (Lewinska et al. 2014b) to analyze both general and specific susceptibility to DNA damage (here breaks) among *C. albicans* chromosomes during long-term culture (Fig. 5).

As chromosome R and chromosome I may migrate together, DNA breaks and replication intermediates were analyzed in joined R + I fraction and six separate fractions, namely chromosome II, chromosome III, chromosome IV, chromosome V, chromosome VI and chromosome VII fractions (Fig. 5a). After 90 days of long-term culture in control conditions, the most susceptible to DNA breaks were chromosomes III (haploid and tetraploid cells) and IV (diploid cells); an increase of about 13 to 14% in DNA breaks was observed, respectively (data not shown). Stress stimuli also promoted chromosomal DNA breaks, but various chromosomes were involved, e.g. chromosomes R, I and III in haploid cells, chromosomes VI and VII in diploid cells and chromosomes II, V and VII in tetraploid cells (data not shown). A joined analysis of chromosomal DNA breaks (Fig. 5b) revealed a statistically significant increase in chromosomal DNA breaks after 90 days of culture of haploid, diploid and tetraploid cells in control conditions and upon treatment with stress stimuli compared to control conditions after 14 days of culture (Fig. 5b). However, stress agents did not potentiate the levels of DNA breaks compared to control conditions after 90 days of culture (Fig. 5b). Moreover, we would like to verify if replication intermediates may accumulate during long-term culture that would be a sign of replication stress (Adamczyk et al. 2016). In contrast to long-term culture-induced chromosomal DNA breaks (Fig. 5b), an increase in replication intermediates was not observed after 90 days of culture (Fig. 5b). Stress agents also did not promote the accumulation of replication intermediates (Fig. 5b).

### Multivariate data analysis

**Fig. 6:**
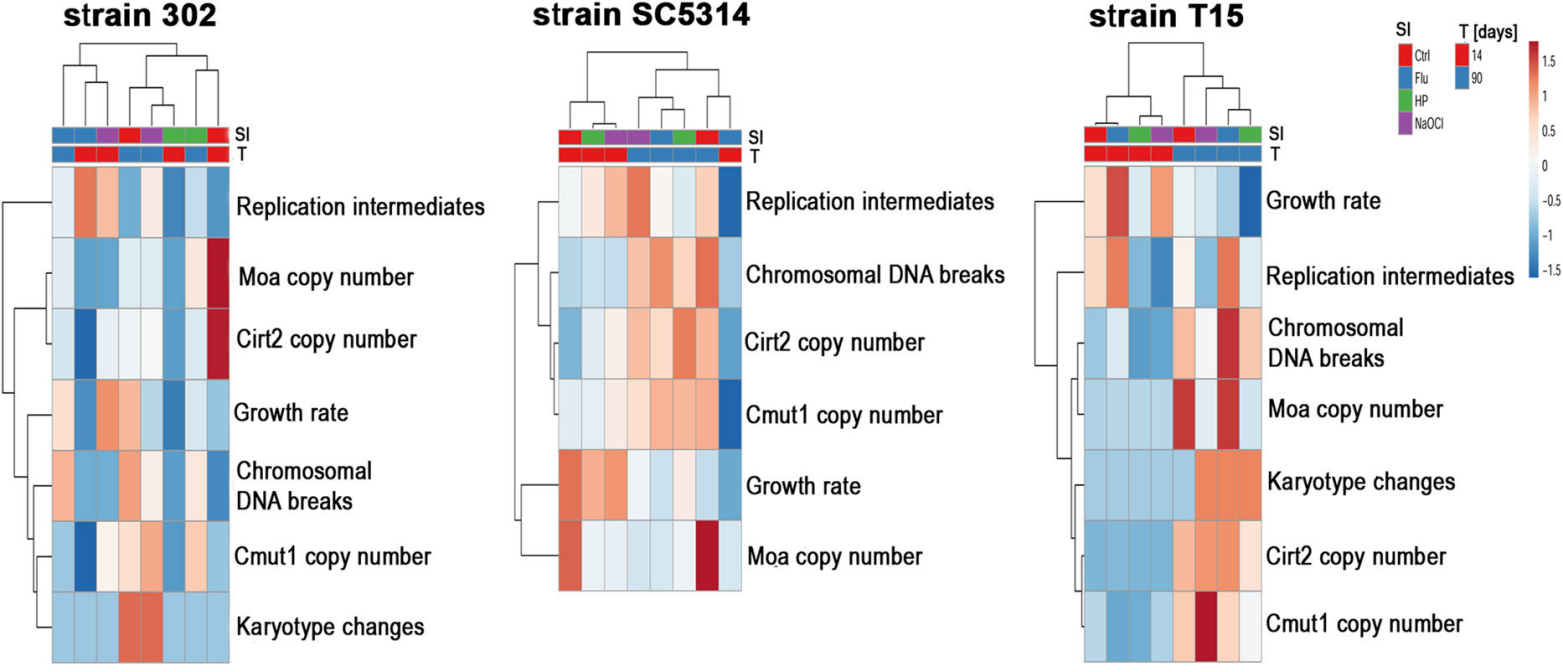
A joined clustering analysis of growth rate, TE copy number, karyotype patterns, chromosomal DNA breaks and replication intermediates as a function of culture duration (14 days of culture versus 90 days of culture) and treatment with stress agents (fluconazole, hydrogen peroxide, hypochlorite). A heat map generated from growth rate, TE copy number, karyotype patterns, chromosomal DNA breaks and replication intermediates data is shown. Hierarchical clustering was created using ClustVis, a web tool for visualizing clustering of multivariate data (BETA) (https://biit.cs.ut.ee/clustvis/). Both rows and columns are clustered using correlation distance and average linkage for HCA analysis. Ctrl, control conditions; SI, stress inductors; Flu, fluconazole; HP, hydrogen peroxide; NaOCl; sodium hypochlorite; T, time; 14, 14 days of culture; 90, 90 days of culture; 302, haploid strain; SC5314, diploid strain; T15, tetraploid strain

We have then considered hierarchical cluster analysis (HCA) using experimental data concerning growth rate, TE copy number, karyotype patterns, chromosomal DNA breaks and replication intermediates as a function of culture duration and treatment with stress agents (Fig. 6). Three *C. albicans* strains, namely haploid, diploid and tetraploid cells were analyzed separately (Fig. 6).

In a case of tetraploid T15 strain, all analyzed experimental parameters were grouped according to culture duration (14 days vs. 90 days) (Fig. 6). No effects of stress stimuli on data clustering were revealed (Fig. 6). Except of fluconazole treatment and 14 days of culture, similar observation was obtained for diploid SC5314 strain (Fig. 6). Again, no effects of stress stimuli on data clustering were noticed (Fig. 6). In contrast, in a case of haploid 302 strain, data clustering according to stress stimuli was revealed and the effect of culture duration was less evident (Fig. 6). This suggests that ploidy state of *C. albicans* strains may modulate adaptive responses during long-term culture, namely in diploid and tetraploid cells, changes in TE activity reflected culture duration, whereas in haploid cells, the effect of stress stimuli on TE activity was much more pronounced.

## Discussion

**Fig. 7:**
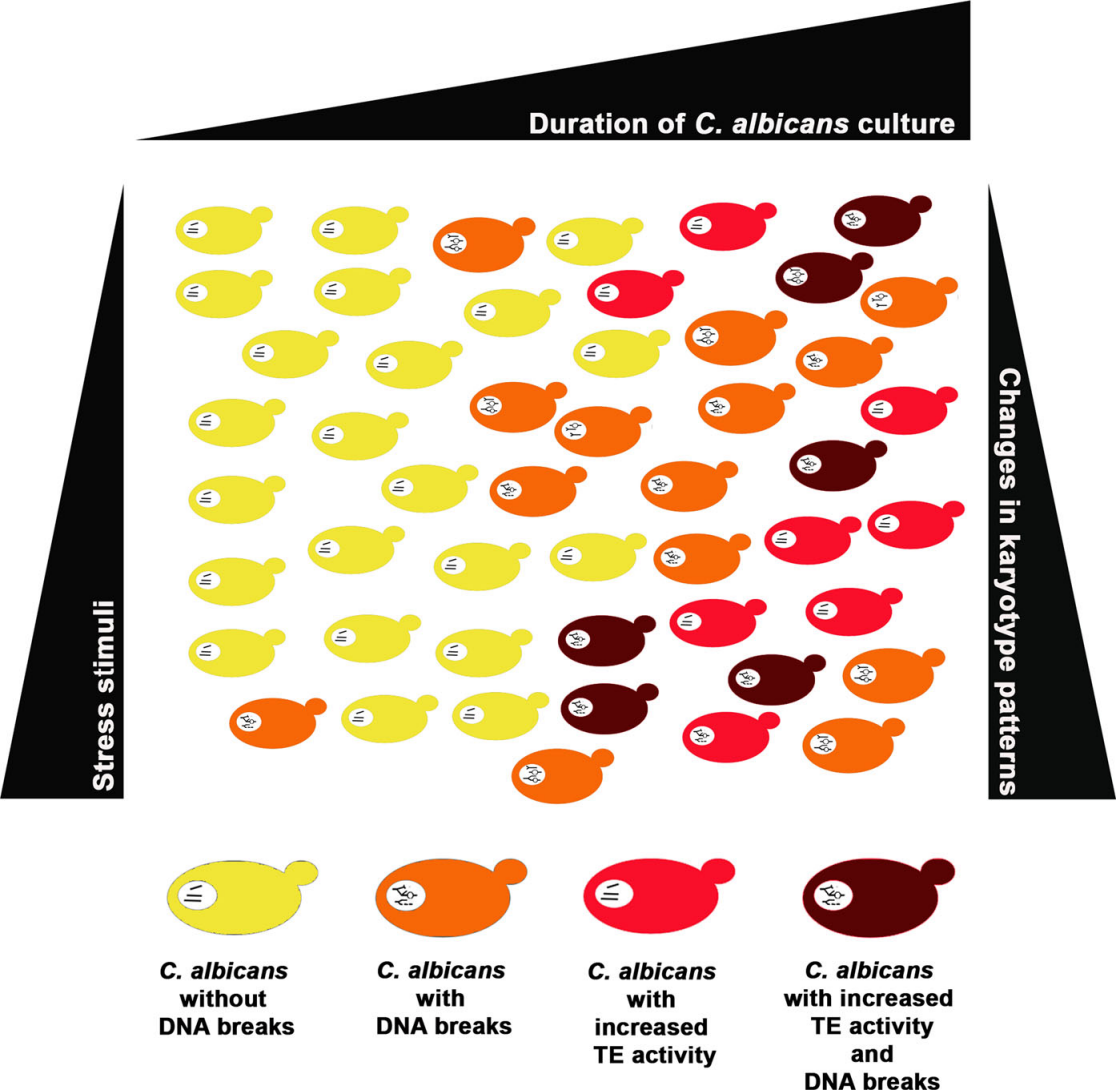
The activity of selected transposable elements, namely Cirt2, Moa and Cmut1 is increased during long-term culture of *C. albicans* cells of different ploidy that is accompanied by variations in karyotype patterns. Stress stimuli (fluconazole, hydrogen peroxide, hypochlorite) did not potentiate TE activity. An increase in chromosomal DNA breaks was also noticed during long-term culture of *C. albicans* cells, however this was not correlated with elevated TE activity. Long-term culture-mediated increase in TE activity and changes in karyotype profiles may in turn induce genomic diversity and cellular heterogeneity of *C. albicans* cell populations

In the present study, we have shown for the first time that the activity of Cirt2, Moa and Cmut1 transposable elements (TEs) is elevated during long-term culture of *C. albicans* cells of different ploidy that is accompanied by changes in chromosome patterns (the occurrence of additional chromosome bands after 90 days of culture both in control conditions as well as after treatment with stress stimuli, namely fluconazole, hydrogen peroxide, hypochlorite) (Fig. 7).

Long-term culture-induced chromosomal DNA breaks were also observed, but the most accented increase in TE activity in T15 tetraploid cells did not correlate with the most pronounced increase in chromosomal DNA breaks in T15 tetraploid cells. In contrast, the levels of long-term culture-mediated chromosomal DNA breaks were comparable among *C. albicans* strains of different ploidy and diverse magnitude of increase in TE activity. Observed genomic changes at chromosomal level may promote genomic diversity and plasticity and drive cellular heterogeneity as an adaptive response during long-term culture of *C. albicans* cell populations.

According to our knowledge, there are no data on changes in the mobilome and genetic stability during long-term culture or replicative aging or chronological aging in *C. albicans.* However, it has been shown that retrotransposition is associated with genome instability during chronological aging in the budding yeast *S. cerevisiae* (Maxwell et al. 2011). Age-related loss of heterozygosity and chromosome loss were limited by mutations or treatments that decreased Ty1 retrotransposition and Ty1 mobility was increased in old yeast cells, and new retro-mobility events were associated with chromosome rearrangements (Maxwell et al. 2011). The authors concluded that retrotransposition may contribute to genetic damage during aging in diverse organisms (Maxwell et al. 2011). Preferential retrotransposition in yeast mother cells has been also found to be correlated with increased genome instability during replicative aging in *S. cerevisiae* (Patterson et al. 2015). Age-related increase in the activity and mobility of transposable elements has been also reported in mammalian cells and tissues (De Cecco et al. 2013a, b; Colombo et al. 2018), however, no conclusive biological role(s) of these events have been provided. It has been observed that during normal aging several families of retrotransposable elements (RTEs) are transcribed in mouse tissues and in advanced age the expression culminates in active transposition (De Cecco et al. 2013b). The authors suggested that the mobilization of RTEs is likely to be an important contributor to the progressive dysfunction of aging cells (De Cecco et al. 2013b). The expression of transposable elements was also activated during replicative senescence and stress-induced senescence (5-azacytidine, hydrogen peroxide and adriamycin treatments) in human fibroblasts (Colombo et al. 2018). The authors concluded that the expression of TEs might play a role in immune mediated clearance of senescent cells (Colombo et al. 2018). More recently, it has been reported that during cellular senescence, LINE-1 retrotransposable elements became transcriptionally de-repressed and activated a type-I interferon (IFN-I) response that promoted age-associated inflammation (De Cecco et al. 2019).

Moreover, information on molecular mechanisms that regulate replicative lifespan (RLS) and chronological lifespan (CLS) or survival and adaptive responses during long-term culture of *C. albicans* is scarce and fragmentary (Fu et al. 2008; Li et al. 2011; Chen et al. 2012; Gil-Bona et al. 2016). *SIR2* gene, a member of the sirtuin family of genes, has been recognized as a regulator of replicative lifespan in *C. albicans* (Fu et al. 2008). Increased *SIR2* gene dosage extended RLS, whereas knockout of *SIR2* gene caused a decrease of RLS (Fu et al. 2008). Replicative aging in clinical *C. glabrata* populations has been also investigated (Bouklas et al. 2017). Old *C. glabrata* cells were found to be more resistant to hydrogen peroxide and neutrophil killing, whereas young cells adhered better to epithelial cell layers and the virulence of old compared to younger *C. glabrata* cells was enhanced in the *Galleria mellonella* infection model (Bouklas et al. 2017). The authors concluded that the pathogenesis may be affected by the generational age distribution of the infecting *C. glabrata* population in a host and replicative aging may play an unanticipated role in the transition from a commensal to a pathogen state (Bouklas et al. 2017). Two proteins, namely Goa1p, a regulator of the *C. albicans* mitochondrial complex I, and the cell wall protein Ecm33 have been documented to modulate CLS (Li et al. 2011; Gil-Bona et al. 2016). Deletion of *GOA1* (growth and oxidant adaptation) gene resulted in oxidative stress, apoptosis and decreased CLS (Li et al. 2011) and caloric restriction restored CLS of the *goa1* null mutant of *C. albicans* (Chen et al. 2012). *C. albicans ecm33/ecm33* mutant devoid of a glycosylphosphatidylinositol-anchored protein involved in fungal cell wall integrity has been also shown to be hypersensitive to temperature, osmotic and oxidative stresses and to have a shortened CLS compared to wild-type strain (Gil-Bona et al. 2016). Oxidative stress is also implicated in chronological aging in other *Candida* species, namely *C. glabrata* as *C. glabrata* cells lacking both superoxide dismutases Cu,ZnSOD (Sod1) and MnSOD (Sod2) showed a high rate of spontaneous mutation and decreased CLS compared to wild-type strain (Briones-Martin-del-Campo et al. 2015). During chronological aging, yeast cells are subjected to nutritional stress and oxidative stress due to the fact that yeast cells are maintained in a spent SC medium (Longo et al. 2012). Moreover, CLS is limited by the accumulation of acetic acid in a spent SC medium that may induce cell death itself and affect CLS results (Burtner et al. 2009). Indeed, more recently, a number of potential artifacts that can affect CLS results obtained and their interpretation has been highlighted (Longo et al. 2012). Thus, to study long-term culture-mediated effects on *C. albicans* mobilome and genetic stability, we decided to use a rich YPD medium that was replaced every 2 days by a fresh one during 90 days of culture to limit nutritional stress- and oxidative stress-mediated effects on cell viability and related results. Indeed, no significant changes in cell viability were observed during 90 days of culture in control conditions (this study).

It has been repeatedly reported that the expression of transposable elements is induced by stress conditions in yeasts, plants, animals and human (McClintock 1984; Chen et al. 2003; Hashida et al. 2003; Todeschini et al. 2005; Hashida et al. 2006; Sehgal et al. 2007; Chenais et al. 2012; Jardim et al. 2015; Miousse et al. 2015; Morales et al. 2015). The mobility of TEs can be considered as a double-edged sword (Chenais et al. 2012). It may promote the occurrence of deleterious mutations, gene disruption and chromosome rearrangements, but on the other hand, transposition activity may be also beneficial and the mutational potential of TEs may contribute to the genetic diversity of organisms and genetic adaptations to stressful environments (Chenais et al. 2012). Indeed, transposon integration may enhance the expression of stress response genes and may drive adaptation to stress conditions (Feng et al. 2013; Esnault et al. 2019). The integration preference of Tf1, a long-terminal repeat retrotransposon in *Schizosaccharomyces pombe*, for the promoters of stress response genes has been documented (Feng et al. 2013) and the authors suggested that the ability of Tf1 to enhance the expression of these genes co-evolved to promote the survival of yeast cells under stress (Feng et al. 2013). In our experimental conditions, increased TE activity (increased TE copy number) was more related to culture duration (90 days) than initial stimulation with stress stimuli as stress stimuli did not potentate TE activity compared to control conditions after 90 days of long-term culture (this study). In general, this observation may reflect age-related changes in TE activity and associated mechanisms, namely the loss of histones or heterochromatin or aberrant RNAi pathway (Hu et al. 2014; Orr 2016; Drinnenberg et al. 2009). However, more studies are needed to reveal the molecular basis of long-term culture-mediated changes in TE activity in *C. albicans*.

Except of stable karyotype profile of diploid cells, affected TE activity correlated with the occurrence of additional chromosome bands in haploid and tetraploid cells both in control conditions and after treatment with stress agents that confirm the genomic plasticity of *Candida* species (Selmecki et al. 2010; Berman 2016; Todd et al. 2017) and may be a part of adaptive response during long-term culture. Haploid, diploid and tetraploid *Candida* cells can be observed as well as aneuploidy may be promoted (Perepnikhatka et al. 1999; Hickman et al. 2013; Selmecki et al. 2005) as a result of drug-induced genomic instability or as a selective advantage in the presence of the drug (Harrison et al. 2014). Variations in karyotype profiles and changes in ploidy state may drive phenotypic changes e.g., adaptations to stressful environments, host niches and antifungal drug treatments (Selmecki et al. 2010; Berman 2016). Several mechanisms that underlie the genomic diversity of *C. albicans* have been proposed, e.g., chromosome length polymorphism, reciprocal translocation at the major repeat sequence loci, chromosomal deletion and trisomy of individual chromosomes (Chibana et al. 2000). For example, azole resistance in *C. albicans* is accompanied by a specific segmental aneuploidy (an isochromosome that is a fusion of the two left arms of chromosome V (Selmecki et al. 2006) that resulted in the amplification of two fluconazole resistance genes *ERG11* and *TAC1* (Selmecki et al. 2008). Moreover, trisomy of chromosome R and trisomy of chromosome IV may also confer resistance to azole treatment in *C. albicans* (Li et al. 2015; Anderson et al. 2017).

In summary, an adaptive response during long-term culture of *C. albicans* cells of various ploidy states has been documented that is based on increased TE activity and changes in karyotype patterns. Long-term culture promoted chromosomal DNA breaks, however, genetic instability did not affect cell viability. Stress conditions (fluconazole, hydrogen peroxide and hypochlorite treatments) have less pronounced effect on TE activity than culture duration (90 days). Shifts in karyotype profiles may in turn promote genomic plasticity and cellular heterogeneity that may modulate cell fitness and lifespan and result in the selection of best adapted cells within a *C. albicans* cell population.
